# Retrospective assessment of patient characteristics and healthcare costs prior to a diagnosis of Alzheimer’s disease in an administrative claims database

**DOI:** 10.1186/s12877-018-0920-2

**Published:** 2018-10-16

**Authors:** Radhika Nair, Virginia S. Haynes, Mir Siadaty, Nick C. Patel, Adam S. Fleisher, Derek Van Amerongen, Michael M. Witte, AnnCatherine M. Downing, Leslie Ann Hazel Fernandez, Vishal Saundankar, Daniel E. Ball

**Affiliations:** 1 Comprehensive Health Insights, Louisville, USA; 2 Eli Lilly and Company, Indianapolis, USA; 3 Humana Inc., Louisville, USA; 4 Lilly Corporate Center, Drop Code 1730, Indianapolis, IN 46285 USA

**Keywords:** Alzheimer’s disease, Comorbidities, Costs

## Abstract

**Background:**

The objective of this study was to examine patient characteristics and health care resource utilization (HCRU) in the 36 months prior to a confirmatory diagnosis of Alzheimer’s disease (AD) compared to a matched cohort without dementia during the same time interval.

**Methods:**

Patients newly diagnosed with AD (with ≥2 claims) were identified between January 1, 2013 to September 31, 2015, and the date of the second claim for AD was defined as the index date. Patients were enrolled for at least 36 months prior to index date. The AD cohort was matched to a cohort with no AD or dementia codes (1:3) on age, gender, race/ethnicity, and enrollment duration prior to the index date. Descriptive analyses were used to summarize patient characteristics, HCRU, and healthcare costs prior to the confirmatory AD diagnosis. The classification and regression tree analysis and logistic regression were used to identify factors associated with the AD diagnosis.

**Results:**

The AD cohort (*N* = 16,494) had significantly higher comorbidity indices and greater odds of comorbid mental and behavioral diagnoses, including mild cognitive impairment, mood and anxiety disorders, behavioral disturbances, and cerebrovascular disease, heart disease, urinary tract infections, and pneumonia than the matched non-AD or dementia cohort (*N* **=** 49,482). During the six-month period before the confirmatory AD diagnosis, AD medication use and diagnosis of mild cognitive impairment, Parkinson’s disease, or mood disorder were the strongest predictors of a subsequent confirmatory diagnosis of AD. Greater HCRU and healthcare costs were observed for the AD cohort primarily during the six-month period before the confirmatory AD diagnosis.

**Conclusion:**

The results of this study demonstrated a higher comorbidity burden and higher costs for patients prior to a diagnosis of AD in comparison to the matched cohort. Several comorbidities were associated with a subsequent diagnosis of AD.

**Electronic supplementary material:**

The online version of this article (10.1186/s12877-018-0920-2) contains supplementary material, which is available to authorized users.

## Background

Development of Alzheimer’s disease (AD) occurs more frequently in the elderly. With an increasing elderly population in the U.S., the prevalence and associated healthcare costs of AD are expected to rise significantly in the absence of any intervention or medication to slow or stop cognitive and functional decline in these patients [1]. Many of the compounds in development to address this issue aim to target the underlying AD pathophysiology, such as modulation of amyloid and tau protein deposition, and therefore may have the potential to slow the progression of disease. In anticipation of this potential shift away from the treatment paradigm of the currently available AD-indicated medications, which are mainly used for symptom management, the ability to accurately identify individuals earlier in the course of disease, prior to irreversible neuronal dysfunction, becomes critical [2, 3]. Therefore, clinical tools are needed to help identify patients earlier in the spectrum of illness and facilitate the ongoing development of disease modifying agents with the ability to alter AD progression.

For research studies that utilize administrative claims data, current diagnostic codes make it challenging to identify patients with AD until the later stages of the illness and require unique approaches to study trends before diagnosis occurs. Prior studies have used various methods to delineate how patients with AD are identified. These include, prospective observational cohorts, [4] using samples derived from electronic medical records of family practitioners, [5] Medicare databases, [6] Medicaid databases, [7] and commercial managed healthcare databases [8–10].

Among the studies utilizing administrative data, Gilden et al. [6] identified four major pathways that led to an AD diagnosis among Medicare Fee For Service (FFS) beneficiaries: AD as the initial diagnosis or cognitive disturbance followed by AD; dementia with suspected etiologies, followed by AD; dementia without known cause, followed by AD; and a triple pathway which included cognitive disturbance followed by dementia of unknown cause, followed by AD. Jaakkimainen et al. [5] described an algorithm of “one hospitalization code or three physician claims codes at least 30 days apart in a two year period OR a prescription filled for an Alzheimer’s disease and related dementias (AD-RD) specific medication” with high sensitivity, specificity, and positive predictive value to identify AD-RD. These studies highlight the challenge of identifying patients with AD from administrative claims data.

Along with the identification of patients with AD, several studies have aimed to characterize the management of these patients prior to diagnosis. In the 12–18 month period prior to the initial diagnosis of AD, patients tend to have increases in healthcare resource utilization (HCRU) and healthcare costs [6, 9, 11]. A study by Gilden et al. (2015) [6] demonstrated a rapid increase in total monthly Medicare expenditures shortly before AD diagnosis, followed by a rapid decline in expenditures. In both Medicare and Medicaid populations, individuals with AD or AD-RD incurred higher expenditures than matched controls in the 12 months prior to diagnosis [7, 9, 11]. A majority of these increases have been attributed to outpatient services, inpatient, and acute care services [7, 11].

The objective of the current study was to understand the pre-diagnostic journey of a cohort of patients who were newly diagnosed with AD. The characterization of this pre-diagnostic journey of patients with AD builds on previous research through the examination of clinical characteristics, socioeconomic attributes, and behavioral characteristics. In addition, this study captures HCRU and costs during the 36 months prior to a confirmatory AD diagnosis in comparison to a matched cohort without AD or dementia. With a longer window of examination prior to a confirmed diagnosis of AD, the current study also aimed to identify potential indicators available in administrative claims data that may help to predict patients who will be subsequently diagnosed with AD.

## Methods

This was a retrospective, observational study using a linked database comprised of information from two databases, retrospective claims and the AmeriLINK data provided by Knowledge Base Management (KBM). The claims data includes billing for inpatient and outpatient office visits and outpatient prescription medication filled for millions of participants. The data includes claims for patients enrolled in commercial or Medicare plans. For the purposes of this study, we included individuals enrolled in commercial or Medicare Advantage and Prescription Drug plans (MAPD) with both medical and pharmacy coverage. Medicare Advantage plans are insurance plans offered to consumers through private companies that cover medical and hospital services that are included under Medicare parts A and B and include additional coverage not available in Medicare, typically including a prescription drug plan. The US federal government reimburses private companies approved to sell Medicare Advantage plans for those services covered in Medicare parts A and B. The consumer’s premium covers additional services and benefits that are unique to the Medicare Advantage Plan [12].

All medical and pharmacy claims included in the study are fully adjudicated and paid. The enrollment, medical, and pharmacy claims data of individuals enrolled in the MAPD were linked using a unique identifier to three variables from AmeriLINK data from KBM. AmeriLINK data consists of consumer, census, and computed behavioral data using publicly available information (i.e., public records), retail transaction data (i.e., credit card purchases), and computed variables derived from census-type information and/or the combination of data to generate new variables. For this study we included three variables from AmeriLINK namely, estimated household income, percent 2010 white collar and blue collar employed (the percentage of the population in the census-area employed in a white collar or blue collar industry). The study protocol that included a description of the research database and methods was reviewed and approved by an external institutional review board.

**Fig. 1:**
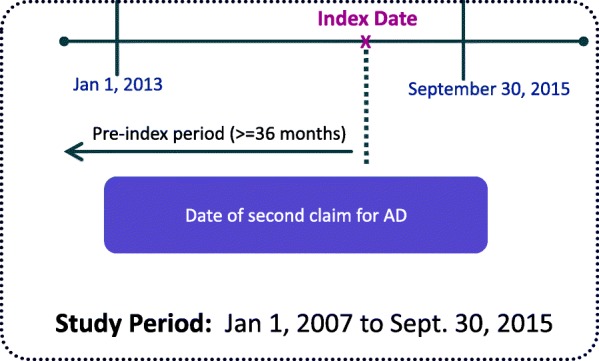
Study Design

Patients 55–89 years of age with two or more claims for AD [International Statistical Classification of Diseases, Ninth Revision, Clinical Modification (ICD-9-CM) 331.0×] on different dates within 18 months of each other between January 1, 2013 and September 30, 2015, were identified (Fig. 1). The date of the second claim for AD was set as the index date. Patients were required to have continuous enrollment for at least 36 months prior to the index date, and had no medical claims with diagnosis codes for AD during the pre-index period (with the exception of the first medical claim for AD).

This AD cohort was matched at the individual patient level with a cohort during the same time interval with no AD or dementia codes (matched cohort) on age (same age in years), gender, race/ethnicity, and enrollment duration prior to the index date (± 3 months). Each individual patient in the AD cohort was matched based on demographics with three individual patients from the control group of patients without any AD or dementia codes. The cohorts were matched only on demographics to allow for a clearer evaluation of the patient’s clinical characteristics prior to the diagnosis of AD, while minimizing any demographic differences.

AD is a form of dementia; therefore, it is likely that patients with a diagnosis of dementia, might actually have AD. To ensure our matched cohort did not have undiagnosed AD dementia, we required that they have no diagnosis codes for dementia. Conversely, for the AD cohort, it is possible that physicians would document dementia without confidence in classifying as AD, so we did not want to exclude patients with two diagnoses of AD dementia due to the presence of a less specific dementia code.

This matched non-AD or dementia cohort were required to have a medical claim within 30 days of the index date of the matched patient from the AD cohort and no medical claims with diagnosis codes for AD, mild cognitive impairment (MCI; ICD-9-CM: 331.83), or AD-related or unrelated dementia codes for the entire length of enrollment. The index date for the matched patient was the date of their medical claim which was within 30 days of their matched AD cohort member’s second diagnosis.

### Variables

Medical and pharmacy claims data, along with AmeriLINK data, were examined prior to the confirmatory AD diagnosis in order to understand demographic, clinical, and socioeconomic/behavioral characteristics of these patients. Prior to the confirmatory diagnosis of AD, baseline demographics including age, gender, race/ethnicity (MAPD only), region of residence, insurance type (MAPD or commercial), and low income subsidy status were evaluated. Low income subsidy status refers to patients with limited resources and an income below 150% of the U.S. federal poverty threshold who were eligible for additional premium and cost-share assistance for prescription drugs. Patients eligible for both Medicare and Medicaid (dual eligible) were also identified.

Comorbidities were evaluated using the Deyo Charlson Comorbidity Index (DCCI) score [13–15] and the RxRisk-V Score [16]. The DCCI score is based on 17 categories of comorbidities, which are used to calculate a score that reflects the cumulative increased likelihood of one-year mortality [13]. The evolution of the Deyo-Charlson methodology has permitted researchers to use the score as an assessment of overall patient health risk. The RxRisk-V Score [16] is a pharmacy-based comorbidity index that involves the identification of 45 distinct medical condition categories via their associated medication treatments. Of the 45 conditions, three that are defined based on claims for durable medical equipment (neurogenic bladder, ostomy, and urinary incontinence) were not included in this study given these claims are not captured in pharmacy claims data.

The prevalence of pre-specified chronic comorbidities and the proportions of patients with annual wellness visits and cognitive assessments prior to the diagnosis of AD were compared between the AD cohort and the matched cohort. The proportion of patients who filled prescriptions for AD medications [cholinesterase inhibitors (donepezil, galantamine, rivastigmine) or N-methyl-D-aspartate (NMDA) antagonist (memantine)] during the pre-index period was also compared across both cohorts.

To evaluate trends in utilization, HCRU and healthcare costs were measured at 6-month intervals prior to the confirmatory diagnosis of AD. Medical HCRU included the number of outpatient, inpatient, and emergency department (ED) visits. Pharmacy HCRU included the calculated number of unique medication classes filled by a patient. The total costs (paid by plan and patient) included medical (outpatient, inpatient, and ED), pharmacy, and total healthcare costs (medical plus pharmacy); these costs were adjusted to 2015.

Socioeconomic and behavioral characteristics play an important role in patient behavior related to the consumption of health care. As such, we used variables identified from the AmeriLINK data including household income, occupation in the census area where the patient resided (blue collar vs. white collar), and education. Furthermore, we used behavior segmentation developed by Humana that provides insights on how individuals naturally group themselves based on their general propensity to engage in their health/healthcare system and with the health plan. The behavior segmentation includes multiple distinct behavioral groups, and are further classified into whether individuals have chronic health issues (chronic) or not (healthy). At the time of this study, the behavior segmentation was applied only to the MAPD population; however, the number of patients in this study enrolled in a commercial plan was low (1.0%). The behavior segmentation was included in the analyses to control for individual variations, but the results for the segments themselves are not provided due to their proprietary nature.

### Analyses

Descriptive analyses were used to compare the pre-index demographic, clinical, and socioeconomic/behavioral characteristics of the AD and matched cohorts. HCRU and healthcare costs for the 0–36 months prior to the index date were analyzed for both cohorts. Mean and median cost components were computed, and mean cost components were compared across groups using t-test.

The associations between patient characteristics and AD diagnosis were tested using multivariate logistic regression models. Given the large number of variables and uncertainty as to their potential interactions, a classification and regression tree (CART) [17, 18] method was used to refine the selection of variables for inclusion in the logistic regression model. The CART analysis is a decision tree method that uses recursive partitioning of data into strata, enabling a study population to be categorized into meaningful subsets. The advantages of using CART are that it is a non-parametric technique not dependent on assumptions regarding distribution of the variables in a dataset, and it can be used to assess both dichotomous and continuous outcome variables [18].

In addition to the selection of variables for inclusion, the CART analysis further aided in identifying the most important explanatory variables in the dataset, which were most predictive of a diagnosis of AD. The top 15 explanatory variables from the CART analysis, along with their two-way interactions, were included in the logistic regression models.

For the final logistic regression model, the forward selection method was used with entry criteria of *P* ≤ 0.05 and retention criteria of *P* ≤ 0.2. In the results for the regression analysis, the ratios of odds ratios (RORs) are reported for interactions. The RORs is an estimate derived from dividing one odds ratio (OR) by another (i.e., OR X/OR Y) when calculating the interaction of one factor with another. In the logistic regression models, the ROR is the exponent of the beta of the two-way interaction term. For example, variable X has OR of 50 and variable Y has OR of 100; both ORs are larger than the neutral value of 1. The ratio of OR X/OR Y is 50/100, or 0.5; this ROR is smaller than 1; however, having an ROR less than 1 does not mean the interaction of variables X and Y has led to a decrease in the odds of having the outcome.

## Results

A total of 16,558 patients newly diagnosed with AD were identified, and of these, 16,494 patients were matched with 49,482 patients in the matched cohort (with no AD or dementia-related diagnoses). Patients in both cohorts were 79.9 [standard deviation (SD) 6.1] years old, with a higher proportion of women (59.4%), and of white race (84.1%, Table 1). The majority of patients were enrolled in MAPD (~ 99%), and the average length of pre-index enrollment was 68.9 (SD 19.4) months.

**Table 1:** Demographic, socioeconomic and behavioral characteristics of Alzheimer’s disease and matched cohorts

Characteristic	AD	Matched Cohort (no AD or dementia)	*P* value*
N	16,494	49,482	–
Age in years, mean [SD]	79.9 [6.1]	79.9 [6.1]	0.830
Gender, n (%)			
Female	9800 (59.4)	29,400 (59.4)	1.000
Male	6694 (40.6)	20,082 (40.6)	
Race/ Ethnicity, n (%)			
White	13,879 (84.1)	41,637 (84.1)	1.000
Black	1970 (11.9)	5910 (11.9)	
Hispanic	317 (1.9)	951 (1.9)	
Other	328 (1.9)	984 (1.9)	
Geographic Region, n (%)			
Northeast	328 (1.9)	1081 (2.2)	< 0.001
Midwest	4080 (24.7)	13,227 (26.7)	
South	10,859 (65.8)	30,862 (62.4)	
West	1227 (7.4)	4312 (8.7)	
Plan Type, n (%)			
MAPD	16,391 (99.4)	48,923 (98.9)	< 0.001
Commercial	103 (0.6)	559 (1.1)	
Plan Characteristics (MAPD), n (%)			
Low Income Subsidy Status Only	2566 (15.5)	4531 (9.1)	< 0.001
Dual Eligibility (Medicare and Medicaid) Only	32 (0.2)	61 (0.1)	0.030
Length of Pre-index Enrollment – mean [SD]	68.9 [19.4]	68.9 [19.4]	0.980
Estimated Household Income, n (%)			
< $15,000	3465 (21.0)	10,748 (21.7)	0.003
$15,000–$29,999	2589 (15.7)	7948 (16.0)	
$30,000–$49,999	3433 (20.8)	10,467 (21.1)	
$50,000–$99,999	3749 (22.7)	12,250 (24.7)	
> =$100,000^¥^	1084 (6.6)	3757 (7.6)	
Unknown	2174 (13.2)	4312 (8.7)	
Census 2010 Percent Blue Collar Employed, n (%)			
0–14%	2940 (17.8)	10,086 (20.3)	< 0.001
15–20%	2802 (16.9)	9361 (18.9)	
21–26%	3077 (18.6)	9765 (19.7)	
27–33%	3030 (18.4)	8846 (17.8)	
34–99%	2471 (14.9)	7112 (14.3)	
Unknown	2174 (13.2)	4312 (8.7)	
Census 2010 Percent White Collar Employed, n (%)			< 0.001
0–48%	3149 (19.1)	9305 (18.8)	
49–56%	3167 (19.2)	9505 (19.2)	
57–64%	2981 (18.1)	9620 (19.4)	
65–74%	2875 (17.4)	9406 (19.0)	
75–99%	2148 (13.0)	7334 (14.8)	
Unknown	2174 (13.2)	4312 (8.7)	
Index AD diagnosing Provider, n (%) (*N* = 16,558)			
Primary Care	5800 (35.1)		
Geriatrics	526 (3.2)		
Neurology	2225 (13.5)		
Psychiatry	813 (4.9)		
Psychology	240 (1.5)		
Other	6906 (41.8)		
Multiple Visits to Index AD-diagnosing provider	12,838 (77.5)		
Only one Visit to Index AD-diagnosing provider	3720 (22.5)		
Time between first and second claim with AD diagnosis	Average: 92 days SD: 125 Median: 34 days		

Abbreviations: *AD* Alzheimer’s disease, *MAPD* Medicare Advantage Prescription Drug, *SD* Standard deviation

Cohorts were matched on age, gender, race and length of pre-index enrollment

¥ Categories were merged; *Chi square test used for categorical variables; Wilcoxon rank sum test used for continuous variables; Significance level set at *P* < 0.05

Please note that for identifying most commonly seen provider were identified prior to matching (*N* = 16,558) and we have identified only outpatient visits. The index diagnoses for 35 patients were not at an outpatient facility. The specialties of 15.7% of the index AD-diagnosing providers were unknown because the specialty data were missing from the claims database

Sociodemographically (Table 1), the AD cohort was from communities with fewer white-collar, employed professionals. Based on behavior segmentation, a larger proportion of the AD cohort was classified as having chronic disease (53.3% vs. 37.1%), compared with the matched cohort, and the patterns of behavior regarding their healthcare varied within the classifications of Healthy and Chronic. An index diagnosis for AD was made by the primary care physician in 35.1% of the patients and 77.5% of patients had multiple visits to the index physician prior to index date. The average time between the two claims for AD was 92 [SD 125] days.

A significantly higher proportion of the matched cohort had a claim for an annual wellness visit compared to the AD cohort (35.0% vs. 29.5%, *P* < 0.001, Table 2), but a greater proportion of the AD cohort had cognitive assessments (2.6% vs. 0.2%, *P* < 0.001, Table 2). The majority of patients in the AD cohort (64.0%) had filled a prescription for at least one AD medication during the pre-index period.

**Table 2:** Clinical characteristics of Alzheimer’s disease and matched cohorts

	AD (*n* = 16,494)		Matched Cohort (No AD or Dementia) (*n* = 49,482)		*P* value*
	Mean [SD]	Median	Mean [SD]	Median	
Deyo Charlson Comorbidity Index	3.8 [3.0]	3.0	3.0 [2.9]	2.0	< 0.001
RxRisk-V Score	9.8 [4.0]	10.0	8.3 [3.7]	8.0	< 0.001
Number of Unique Medications Used (Drug Classes)	22.0 [14.0]	20.0	18.2[10.7]	17.0	
Wellness Visit and Assessments, n (%)					
Annual Medicare Wellness Visit	4832 (29.5)		17,132 (35.0)		< 0.001
Cognitive Assessment	435 (2.6)		114 (0.2)		< 0.001
AD medication use at baseline	10,559 (64.0)		884 (1.8)		

Abbreviations: *AD* Alzheimer’s disease, *MCI* Mild Cognitive Impairment, *SD* Standard deviation

RxRisk-V Score was calculated for those with at least one prescription

*Chi square test used for categorical variables; Wilcoxon rank sum test used for continuous variables; Significance level set at *P* < 0.05

The AD cohort had significantly higher DCCI scores (3.8 vs. 3.0, *P* < 0.001) and RxRisk-V Scores (9.8 vs. 8.3, *P* < 0.001) than the matched cohort (Table 2), indicating a higher comorbidity burden. Among the pre-specified comorbidities evaluated, hypertension, dyslipidemia, and other forms of heart disease were the most prevalent in both cohorts (Table 3). Other pre-specified comorbidities with notable differences each occurring more frequently in the AD cohort included mood disorders (+ 22.3% difference), cerebrovascular disease (+ 20.5% difference), urinary tract infection (+ 14.8% difference), anxiety disorder (+ 11.4% difference), and MCI (+ 10.5% difference).

**Table 3:** Prevalence of pre-specified comorbidities for Alzheimer’s disease and matched cohorts

Comorbidity, n (%)	AD (*n* = 16,494)	Matched Cohort (No AD or Dementia) (*n* = 49,482)	*P* value*
Hypertension	14,816 (89.8)	43,198 (87.4)	< 0.001
Dyslipidemia	13,951(84.6)	41,824 (84.6)	0.986
Other forms of heart disease	9434(57.2)	23,597 (47.7)	< 0.001
Osteoarthritis	8372 (50.8)	23,389 (47.3)	< 0.001
Urinary tract infection	8146 (49.4)	17,096 (34.6)	< 0.001
Cerebrovascular disease	7207 (43.7)	11,479 (23.2)	< 0.001
Ischemic heart disease	6963 (42.2)	17,841(36.1)	< 0.001
Diabetes	6866 (41.6)	18,698 (37.8)	< 0.001
Mood disorder	6483 (39.3)	8391 (17.0)	< 0.001
Osteoporosis	5788 (35.1)	16,325(33.0)	< 0.001
Any chronic obstructive pulmonary disease	5728 (34.7)	15,405(31.2)	< 0.001
Acute respiratory infection	5137 (31.1)	16,576 (33.5)	< 0.001
Cancer	4390 (26.6)	14,271(28.9)	< 0.001
Anxiety disorder	4356 (26.4)	7428(15.0)	< 0.001
Heart failure	3957(24.0)	8907(18.0)	< 0.001
Peripheral vascular disease	3547 (21.5)	8155 (16.5)	< 0.001
Atherosclerosis	3435(20.8)	8615(17.4)	< 0.001
Pneumonia	2931(17.8)	5506 (11.1)	< 0.001
Insomnia	2132(12.9)	4704(9.5)	< 0.001
Mild Cognitive Impairment	1727(10.5)	0(0.0)	< 0.001
Diseases of pulmonary circulation	1689 (10.2)	4248 (8.6)	< 0.001
Chronic ulcer of skin	1505(9.1)	2486 (5.0)	< 0.001
Behavioral disturbance	1369(8.3)	0 (0.0)	< 0.001
Venous Thromboembolism	969(5.9)	1999 (4.0)	< 0.001
Parkinson’s disease	872(5.3)	568 (1.1)	< 0.001
Gastric, duodenal, peptic, or gastrojejunal ulcer	854(5.2)	1710(3.5)	< 0.001
Epilepsy	845(5.1)	688(1.4)	< 0.001
Rheumatoid arthritis	771(4.7)	2155 (4.4)	0.087
Lung cancer	233(1.4)	720 (1.5)	0.686

Abbreviations: *AD* Alzheimer’s disease, *MCI* Mild Cognitive Impairment

*Chi square test used; Significance level set at *P* < 0.05

**Fig. 2:**
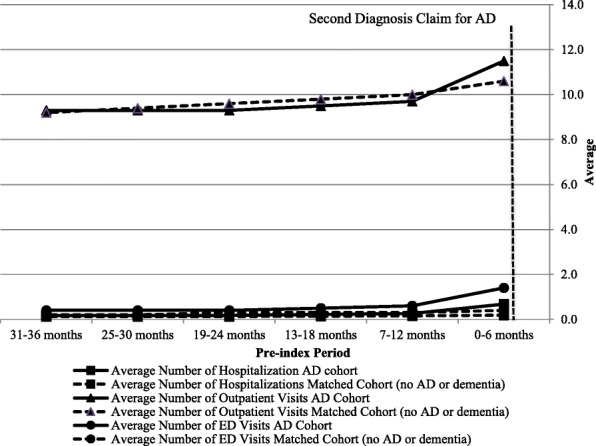
Trends in healthcare resource use per person at six-month intervals for AD and matched cohorts. Abbreviations: AD-Alzheimer's disease, ED-Emergency department

**Fig. 3:**
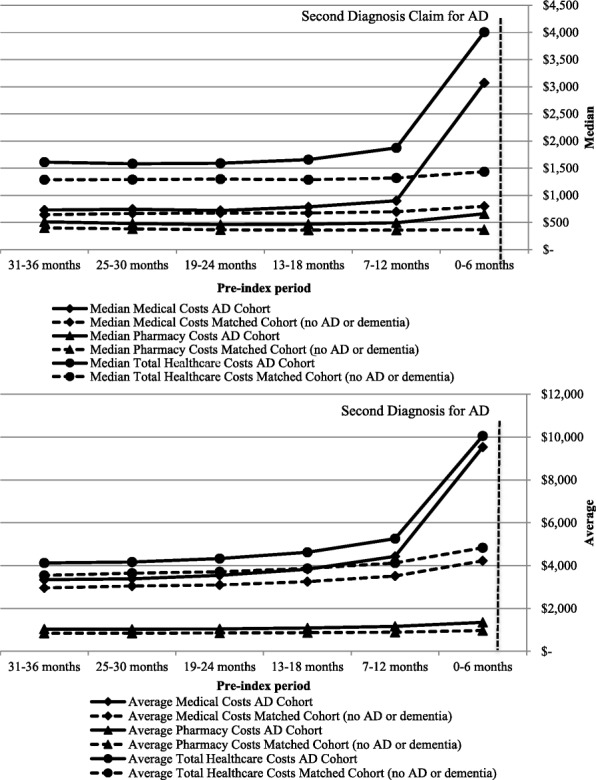
Title: Trends in healthcare cost per person at six-month intervals for AD and matched (No AD or Dementia) cohorts (Adjusted to 2015 dollars). Abbreviations: AD – Alzheimer’s disease

When comparing the average pre-index HCRU (Fig. 2) the number of outpatient visits, hospitalizations, and ED visits at 6-month intervals were similar between the cohorts until six months prior to the index date. For the AD cohort, the number of these visits increased during the six months prior to the index date. A similar trend was observed for healthcare costs, with an increase in mean and median medical costs for the AD cohort occurring during the − 6-month interval prior to the index date (Fig. 3). During this time period, the average total healthcare cost per person was significantly higher for the AD cohort than for the matched cohort ($10,054 vs. $4833, *P* < 0.0001).

**Fig. 4:**
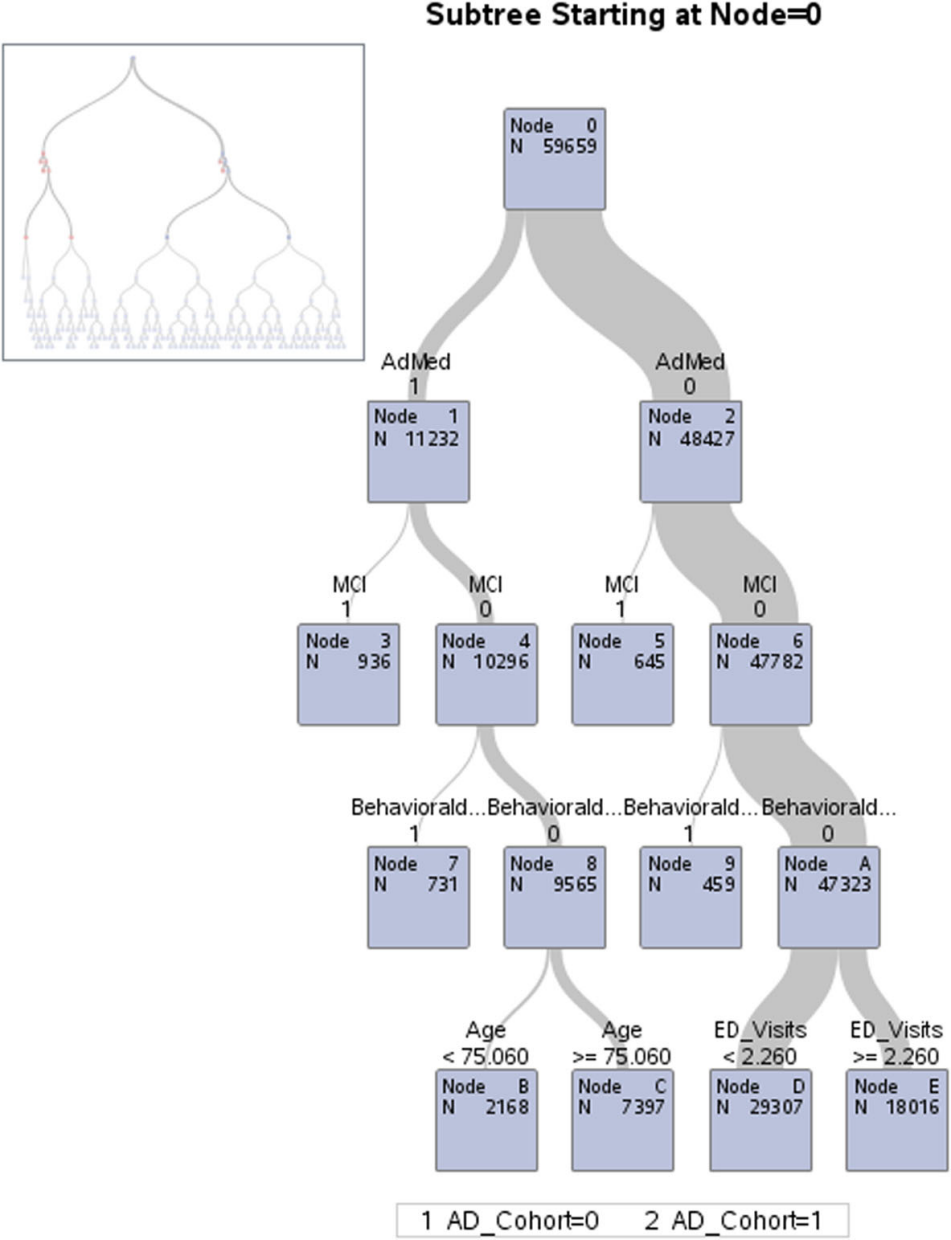
Classification tree model sub-tree graph of factors most predictive of a diagnosis of AD. Abbreviations: AD – Alzheimer’s disease, ED – Emergency Department, MCI – Mild Cognitive Impairment; ADMed – AD medications, Behavioral – Behavioral disturbance Estimates from model controlling for behavioral segmentation.

**Fig. 5:**
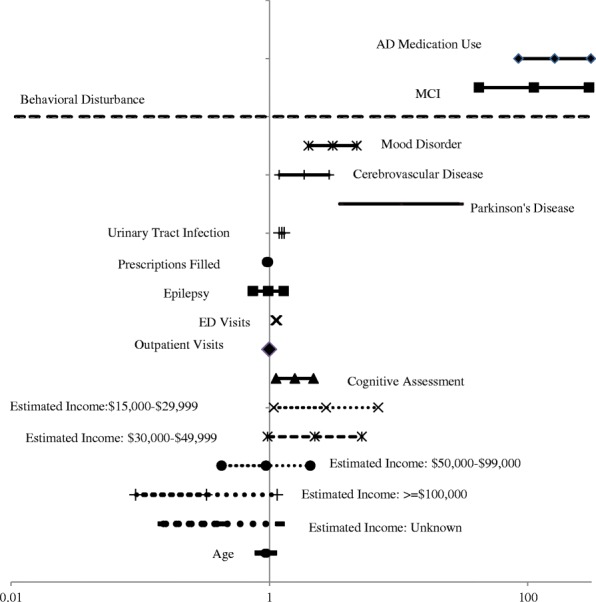
Forest Plot for Logistic Regression: Demographic and Clinical Variables Associated with Diagnosis of AD. Abbreviations: AD – Alzheimer’s disease, ED – Emergency Department, MCI – Mild Cognitive Impairment Estimates from model controlling for behavioral segmentation. The left and right symbol are the lower and upper limits of the 95% confidence interval for the odds of a diagnosis of Alzheimer’s disease

Using the CART analysis, up to 67 potential predictors of an AD diagnosis were evaluated, and the top 15 variables by importance were identified for inclusion in the logistic regression model (Fig. 4). From the tree, we see that use of AD medication is the most important predictor of an AD diagnosis and that an MCI or behavioral disturbance diagnosis, presence of an emergency department visit, and patient age provided additional information to classify patients as likely to receive an AD diagnosis. Due to its size, only the first part of the full tree is illustrated. The model showed excellent specificity (97%) and acceptable sensitivity (77%), and the overall area under the curve was 0.917. The 15 top performing variables and their interactions were used to run a logistic regression model (Fig. 5, Additional file 1: Table S1).

The results of the logistic regression analysis (Additional file 1: Table S1) showed AD medication use had the greatest association with increased likelihood of AD diagnosis (OR = 161.157, *P* < 0.001) followed by the presence of comorbidities such as MCI (OR = 111.626, *P* < 0.001), Parkinson’s disease (OR = 10.081, *P* < 0.001), mood disorder (OR = 3.083, *P* < 0.001), cerebrovascular disease (OR = 1.856, *P* = 0.007), and/or a urinary tract infection (UTI, OR = 1.246, *P* < 0.001) and ED visits (OR = 1.131, *P <* 0.001).

Although there were significant two-way interactions involving AD medication use, ED visit, and mood disorder, estimates at the mean values and distinct categories of interacting variables with these terms were consistently significant and greater than one. Similarly, odds ratios for Parkinson’s disease were consistently greater than one and 5 of the 8 were statistically significant. Thus, the direction of main effects related to AD medication use, ED visit, mood disorder, and Parkinson’s disease can be interpreted. There were no significant two-way interactions with MCI or UTI, facilitating the interpretation of these effects.

The main effect of cerebrovascular disease is not interpretable due to significant two-way interactions and odds ratios that varied in direction. Specifically, the presence of a cerebrovascular disease diagnosis indicated lower likelihood of an AD diagnosis in patients with a cognitive assessment as part of the annual wellness visit (3 of 4 odds ratios significant). Conversely, among patients who did not have a cognitive assessment as part of the annual wellness visit, the cerebrovascular diagnosis indicated a greater likelihood of AD diagnosis (3 of 4 odds ratios significant).

Increasing age (OR = 0.937, *P* < 0.001) decreased the odds of being diagnosed with AD, but this finding should be interpreted with caution as the AD and matched cohorts were matched on age. Additionally, there were significant two way interactions between age and Parkinson’s disease, and between age and cerebrovascular disease. When examining the 96 odds ratios involving age and combinations of values for each of AD Medication use, mood disorder, cerebrovascular disorder, Parkinson’s disease, and income, the majority are significant, 71 were less than one, indicating increased age was associated with lower likelihood of AD and 6 were less than 1. The patient subgroups in which increased age was associated with greater likelihood of AD were those who had not received AD medications and did not have a diagnosis of mood disorder, cerebrovascular disease or Parkinson’s disease and this OR was significant in four of the 6 income categories. The other subgroups in which increased age was associated with greater likelihood of AD were those in the upper two income categories who had not received AD medications, did not have a diagnosis of mood disorder or Parkinson’s disease but did have a cerebrovascular diagnosis.

## Discussion

With our study’s longer duration of time to observe patients’ engagement with the healthcare system, we were able to add to the existing understanding of a patients’ journey prior to confirmatory AD diagnosis. Our study results significantly add to existing literature as the sample of 16,494 patients with a confirmatory diagnosis of AD is notable in terms of its longevity; the median duration of the pre-index period for the AD and matched cohorts was 72 months. In contrast, similar studies by Lin et al. (2016) [11] observed patients 24 months before and after their AD diagnosis, and Gilden et al. (2015) [6] required 12 months of data prior to and following AD diagnosis.

Over a third of the sample received their confirmatory AD diagnosis in the primary care setting, in comparison to less than one fourth receiving this diagnosis from a geriatric or mental health specialist. Also, we found that almost two-thirds of the AD patients received AD-related medications prior to their confirmatory diagnosis, and only 10% of patients in the AD group received an MCI diagnosis prior to their confirmatory diagnosis, all of which may suggest a reluctance to diagnose memory related issues [19]. Some factors that may contribute to the reluctance to make a diagnosis of AD include physician’s decision not to create or increase the emotional stress of a patient. Alternatively, reluctance to make a diagnosis of AD may be reflective of insufficient training or time to confidently make a diagnosis through appropriate assessments resulting in a delayed diagnosis, especially given the ongoing challenge to diagnose based on exclusions [19, 20]. Without a biomarker or pathognomonic test, the diagnosis is always going to be somewhat questionable.

The AD cohort was generally in poorer health than the matched cohort, which was reflected in the significantly higher comorbidity indices and greater odds of comorbid mental and behavioral diagnoses, cerebrovascular disease, and other diseases such as heart disease, UTIs, and pneumonia. This is a similar clinical profile to that observed in the post-index follow-up period by Suehs et al. (2013) [9], with exception of MCI and behavioral disturbance, which were not examined. The presence of various comorbidities in patients with AD highlights the importance that treatment plans for such patients should not be only focused on the expected cognitive decline, but should warrant a multidisciplinary approach to routinely assess for comorbid conditions.

Consistent with other administrative claims studies examining HCRU and cost in the year prior to the first AD diagnosis, [8, 9] this study showed that HCRU for patients with AD was significantly higher than that for the matched cohort. Similarly, Lin and others demonstrated that for patients with AD-RD, from the 5% Medicare sample, HCRU was greater than the control population during the 24 months prior to their first diagnosis, particularly during the most proximal 12 months [11]. This was driven by inpatient, home health, and post-acute care, with virtually no difference in outpatient or physician office visits. In contrast, a prospective study by Zhu et al (2015), [21] that included Medicare beneficiaries who received clinical evaluations for AD every 18 months, found significant differences only in home health and durable medical equipment use. Observed differences in HCRU in these studies could be due to case ascertainment (AD-RD, AD by clinical evaluation, or AD determined based on the presence of two ICD-9 codes), specific study population evaluated (MAPD, a multi-ethnic cohort from northern Manhattan, FFS Medicare), or changes in the approach to medical care of elderly patients over time.

In our study, the total average cost for patients at six months prior to AD diagnosis was greater ($5221 in 2015 dollars) than for the matched cohort. One plausible explanation for this difference may be related to the comorbidity burden. Matching between the AD cohort and the non-AD or dementia cohort was not based on comorbidity, which allowed for a better understanding of the relative comorbidity differences across the cohorts. Additionally, costs associated with the subsequent AD diagnosis are also contributory to the difference. In comparison, the study by Lin et al. identified individuals on the basis of their first recorded AD-RD diagnosis code and found the difference in average pre-diagnosis costs in the six months prior to index was $3571 (in 2014 dollars) [11]. The comparison of AD-RD to control subjects in the 5% Medicare sample included comorbidity in the identification of the matched cohort, supporting the premise that additional costs are incurred as a result of a subsequent AD diagnosis [11].

Our analysis of examining potential predictors of AD is similar to the findings of the study by Jaakkimainen et al. (2016), [5] which showed that the best performing algorithm in Canadian administrative claims data from primary care practices was either a hospitalization or three physician claims separated by 30 days or more in the same 24-month period. The sensitivity and positive predictive value of the algorithm was improved by inclusion of a prescription for a cholinesterase inhibitor. In the CART model, which had good sensitivity and higher specificity, AD medication use, diagnosis of MCI, Parkinson’s disease, and a mood disorder were the strongest predictors of an AD diagnosis.

In extending the interpretation of AD medication use by exploring their interactions with other predictors, we found that the magnitude of association of AD medication use with an AD diagnosis varied, depending on whether patients also had cerebrovascular disease, Parkinson’s disease, mood disorder, or UTI. The odds of AD diagnosis associated with AD medication use was uniformly lower in the subgroups of patients with Parkinson’s disease, UTI, mood disorder or cerebrovascular disease in comparison to patients who were similar on all variables but that particular diagnosis. The predictor of AD medication use may reflect physician and/or patient preference to treat with symptomatic medication prior to receiving a definitive diagnosis, [6] while the most important psychiatric and neurological diagnoses likely reflect disease progression.

The current study’s findings should be interpreted in the light of the following limitations. Characteristic of retrospective, claims-based research, the results may have been influenced by missing data, potential errors in coding, and unmeasured factors, such as psychosocial variables and other clinical variables. In addition, there is an established, multi-factorial gap in diagnosis and observance of AD symptoms. Furthermore, the commonality of the identified comorbidities in the non-AD population may limit the ability to use these as screening measures for potential AD. This study focused on patients with a confirmatory diagnosis for AD and did not include undiagnosed patients or those with only one claim for AD diagnosis. The index date for this study was the confirmatory diagnosis of AD, which meant the costs would include the assessment and other AD-related costs from the first diagnosis of AD. Despite this, our findings are consistent with that of Lin et al., who evaluated costs prior to diagnosis of AD [11]. Additionally, data in this study were obtained from a single health insurance company, and although Humana is a large national health plan with members from various geographic regions, the results may not be generalizable to the overall U.S. population, or to subpopulations within certain geographic regions of the U.S. Moreover, the results may not be generalizable to all Medicare populations due to differences in benefit structure of MAPD and non-MAPD health plans. The matching criteria used in the current study, as well as the method for selecting the AD cohort, may be considered as additional study limitations. We matched the population only on demographic characteristics and not clinical characteristics such as comorbidities. This was so that we can understand the differences in comorbidities between patients, however, these comorbidities may have contributed to increases in healthcare resource use and costs. Additionally, we used second date of claim for AD as index date as opposed to the first. Healthcare resource and costs since the first visit with a claim of AD may have contributed to the increases observed during the six months prior to the second claim with AD. However, in some sensitivity analyses conducted (not shown), the results did not change when first claim for AD was set as index date.

As an administrative claims study, we are only able to associate healthcare resource use with different cohorts defined by diagnostic codes. In the current study, our intent was to understand the total healthcare resource use, so we did not limit to claims specifically related to AD. Other types of observational research that include reason for cost are needed to understand if these increased costs are due to AD.

## Conclusion

Given the growing elderly population and the concomitant increases in prevalence of AD and associated cost of care, it is important to understand the patients’ journey to diagnosis of this disease.

This study demonstrated that prior to the diagnosis of AD, patients had a higher number of comorbidities and incurred higher costs in comparison to a demographically matched cohort. Certain comorbidities that occurred at a higher rate in the AD cohort, namely psychiatric and neurological in nature, may serve as flags to help identify patients most likely to develop AD using administrative claims data. We observed a trend of increasing healthcare costs during the 6-month period prior to the confirmatory diagnosis of AD, offering yet another potential signal that can be gleaned from administrative claims data.

Without a biomarker or a test to detect AD, diagnosis is challenging, since it is essentially a diagnosis of exclusion. This suggests that AD is underdiagnosed, and the true impact of this disease may be greater than what the current study reports. With the pipeline of AD drug development aimed at disease modification, additional research is needed to further understand other clinical presentations that might potentially predict diagnosis of AD more robustly or even earlier than the 36-month-year period used in this study. Furthermore, with early recognition of AD, a better understanding of the stage of illness at which patients are diagnosed with AD, along with these early predictors, can help guide the treatment pathway.

## Additional file

Additional file 1: **Table S1.** Logistic regression model of factors associated with the diagnoses of AD, based on classification tree model. This table lists the parameter estimates, *p*-values, odds ratios, and confidence intervals associated with the logistic regression model based on factors identified in the classification and regression tree analysis. (DOCX 80 kb)

## Abbreviations

AD: Alzheimer’s Disease
AD-RD: Alzheimer’s Disease and Related Dementias
CART: Classification and Regression Tree
DCCI: Deyo Charlson Comorbidity Index
ED: Emergency Department
FFS: Medicare Fee for Service
HCRU: Healthcare Resource Utilization
ICD-9-CM: International Statistical Classification of Diseases, Ninth Revision, Clinical Modification
KBM: Knowledge Base Management
MAPD: Medicare Advantage with Prescription Drug Coverage
MCI: Mild Cognitive Impairment
NMDA: N-methyl-D-aspartate
RORs: Ratios of Odds Ratios
SD: Standard Deviation
UTI: Urinary Tract Infection
