# Supplementary material for: Retrospective assessment of patient characteristics and healthcare costs prior to a diagnosis of Alzheimer’s disease in an administrative claims database
Source: BMC Geriatr. 2018 Oct 16;18:243. doi: 10.1186/s12877-018-0920-2 (PMC6192320; doi:10.1186/s12877-018-0920-2)
Supplement: Supplementary file 1 — Table S1. Logistic regression model of factors associated with the diagnoses of AD, based on classification tree model. This table lists the parameter estimates, p-values, odds ratios, and confidence intervals associated with the logistic regression model based on factors identified in the classification and regression tree analysis. (DOCX 80 kb) [file 12877_2018_920_MOESM1_ESM.docx]

**Supplemental Table: Logistic regression model of factors associated with the diagnoses of AD, based on classification tree model**

| **Parameter/Interactions** | **Value** | | | | **DF** | **Estimate** | **Std Error** | **Wald Chi Sq** | **P Value** | **Odds Ratio /Ratio of Odds Ratios**^†^ | **95% CI: Lower** | **95% CI: Upper** | |
| --- | --- | --- | --- | --- | --- | --- | --- | --- | --- | --- | --- | --- | --- |
| AD Medication Use | Yes | |  | | 1 | 5.082 | 0.330 | 237.746 | <0.0001 | 161.157 | 84.469 | 307.482 | |
| MCI | Yes | |  | | 1 | 4.715 | 0.501 | 88.512 | <0.0001 | 111.626 | 41.798 | 298.137 | |
| Behavioral Disturbance | Yes | |  | | 1 | 8.558 | 44.089 | 0.038 | 0.8461 | 5209.781 | 1.53875E-34 | 1.76391E+41 | |
| ED Visits |  | |  | | 1 | 0.124 | 0.008 | 224.888 | <0.0001 | 1.131 | 1.113 | 1.150 | |
| Mood Disorder | Yes | |  | | 1 | 1.126 | 0.219 | 26.418 | <0.0001 | 3.083 | 2.007 | 4.737 | |
| Prescriptions Filled |  | |  | | 1 | -0.033 | 0.005 | 44.871 | <0.0001 | 0.968 | 0.959 | 0.977 | |
| Cerebrovascular Disease | Yes | |  | | 1 | 0.619 | 0.228 | 7.385 | 0.007 | 1.856 | 1.188 | 2.900 | |
| Parkinson’s Disease | Yes | |  | | 1 | 2.311 | 0.541 | 18.256 | <0.0001 | 10.081 | 3.493 | 29.098 | |
| Urinary Tract Infection | Yes | |  | | 1 | 0.220 | 0.022 | 100.793 | <0.0001 | 1.246 | 1.193 | 1.300 | |
| Outpatient Visits |  | |  | | 1 | -0.005 | 0.000 | 129.864 | <0.0001 | 0.995 | 0.994 | 0.996 | |
| Epilepsy | Yes | |  | | 1 | -0.020 | 0.141 | 0.020 | 0.888 | 0.980 | 0.744 | 1.292 | |
| Cognitive Assessment | Yes | |  | | 1 | 0.453 | 0.170 | 7.057 | 0.008 | 1.573 | 1.126 | 2.196 | |
| Estimated Income^¥^ | $15,000-$29,999 | |  | | 1 | 1.008 | 0.475 | 4.497 | 0.034 | 2.741 | 1.079 | 6.959 | |
| Estimated Income^¥^ | $30,000-$49,999 | |  | | 1 | 0.807 | 0.429 | 3.539 | 0.060 | 2.241 | 0.967 | 5.196 | |
| Estimated Income^¥^ | $50,000-$99,999 | |  | | 1 | -0.062 | 0.403 | 0.024 | 0.878 | 0.940 | 0.426 | 2.072 | |
| Estimated Income^¥^ | >=$100,000 | |  | | 1 | -1.124 | 0.644 | 3.047 | 0.081 | 0.325 | 0.092 | 1.148 | |
| Estimated Income^¥^ | Unknown | |  | | 1 | -0.935 | 0.533 | 3.072 | 0.080 | 0.393 | 0.138 | 1.117 | |
| Age |  | |  | | 1 | -0.065 | 0.007 | 78.529 | <0.0001 | 0.937 | 0.923 | 0.950 | |
| AD Medication Use*Mood Disorder | Yes | | Yes | | 1 | -0.178 | 0.023 | 62.969 | <0.0001 | 0.837 | 0.801 | 0.875 | |
| AD Medication Use*Cerebrovascular Disease | Yes | | Yes | | 1 | -0.090 | 0.023 | 14.924 | 0.000 | 0.914 | 0.874 | 0.957 | |
| AD Medication Use*Cognitive Assessment | Yes | | Yes | | 1 | -0.465 | 0.078 | 36.012 | <0.0001 | 0.628 | 0.540 | 0.731 | |
| AD Medication Use*Parkinson’s Disease | Yes | | Yes | | 1 | -0.269 | 0.057 | 22.177 | <0.0001 | 0.764 | 0.683 | 0.855 | |
| AD Medication Use *Urinary Tract Infection | Yes | Yes | | | 1 | -0.047 | 0.022 | 4.722 | 0.030 | 0.954 | 0.915 | 0.996 | |
| Age*AD Medication Use | Yes |  | | | 1 | -0.042 | 0.004 | 119.777 | <0.0001 | 0.959 | 0.951 | 0.966 | |
| Age*Mood Disorder | Yes |  | | | 1 | -0.012 | 0.003 | 19.855 | <0.0001 | 0.988 | 0.983 | 0.993 | |
| Age*Parkinson’s Disease | Yes |  | | | 1 | -0.023 | 0.007 | 12.249 | 0.001 | 0.977 | 0.965 | 0.990 | |
| Age* Cerebrovascular Disease | Yes |  | | | 1 | -0.008 | 0.003 | 9.588 | 0.002 | 0.992 | 0.987 | 0.997 | |
| ^¥^Estimated Income *Age | $15,000-$29,999 |  | | | 1 | -0.013 | 0.006 | 4.779 | 0.029 | 0.987 | 0.976 | 0.999 | |
| ^¥^Estimated Income *Age | $30,000-$49,999 |  | | | 1 | -0.011 | 0.005 | 4.077 | 0.044 | 0.989 | 0.979 | 1.000 | |
| ^¥^Estimated Income *Age | $50,000-$99,999 |  | | | 1 | 0.000 | 0.005 | 0.003 | 0.953 | 1.000 | 0.990 | 1.010 | |
| ^¥^Estimated Income *Age | >=$100,000 |  | | | 1 | 0.015 | 0.008 | 3.199 | 0.074 | 1.015 | 0.999 | 1.031 | |
| ^¥^Estimated Income *Age | Unknown |  | | | 1 | 0.014 | 0.007 | 4.717 | 0.030 | 1.014 | 1.001 | 1.028 | |
| ED Visits*AD Medication Use | Yes |  | | 1 | | -0.008 | 0.005 | 3.141 | 0.076 | 0.992 | 0.983 | 1.001 | |
| ED Visits*Prescriptions Filled |  |  | | 1 | | -0.001 | 0.000 | 28.629 | <0.0001 | 0.999 | 0.999 | 0.999 | |
| ED Visits*Mood Disorder | Yes |  | | 1 | | -0.007 | 0.003 | 6.067 | 0.014 | 0.993 | 0.988 | 0.999 | |
| Prescriptions Filled*Outpatient Visits |  |  | | 1 | | 0.000 | 0.000 | 25.643 | <0.0001 | 1.000 | 1.000 | | 1.000 |
| Prescriptions Filled* Epilepsy | Yes |  | | 1 | | 0.005 | 0.002 | 4.808 | 0.028 | 1.005 | 1.001 | | 1.010 |
| Prescriptions Filled* Cognitive Assessment | Yes |  | | 1 | | -0.008 | 0.004 | 4.341 | 0.037 | 0.992 | 0.985 | | 1.000 |
| Cerebrovascular Disease *Parkinson’s Disease | Yes | Yes | | 1 | | -0.109 | 0.039 | 8.008 | 0.005 | 0.897 | 0.832 | | 0.967 |
| Cerebrovascular Disease *Cognitive Assessment | Yes | 1 | | 1 | | -0.226 | 0.071 | 10.149 | 0.001 | 0.798 | 0.695 | | 0.917 |
| Mood Disorder *Parkinson’s Disease | Yes | Yes | | 1 | | -0.128 | 0.039 | 10.662 | 0.001 | 0.880 | 0.814 | | 0.950 |
| Cognitive Assessment *Epilepsy | Yes | Yes | | 1 | | -0.341 | 0.120 | 8.121 | 0.004 | 0.711 | 0.563 | | 0.899 |

Abbreviations: AD – Alzheimer’s disease, CI – Confidence Interval, DF – Degrees of Freedom, ED – Emergency Department, MCI – Mild Cognitive Impairment, OR – Odds Ratio, Sq – Square, Std – Standard; Reference group for Estimated income is <$15,000

Behavior segmentation was included in the analyses above to control for individual variations

The ratio of odds ratios (ROR) is an estimate derived from dividing one odds ratio (OR) by another (i.e., OR X/OR Y) when calculating the interaction of one factor with another. In the logistic model, the ROR is the exponent of the beta of the two-way interaction term. Example: Variable X has OR of 50, and variable Y has OR of 100; both ORs are larger than the neutral value of 1. The ratio of ORX/ ORY: 50/100 = 0.5. This ROR (0.5) is smaller than 1. However, having an ROR that is less than 1 does not mean that the interaction of variables X and Y has led to a decrease in the odds of having the outcome.
